# Genomic analysis of putative hybrids between *Potamochoerus* spp. and domestic pigs from sympatric areas in West Africa and Madagascar

**DOI:** 10.1371/journal.pone.0346906

**Published:** 2026-04-21

**Authors:** Rianja Rakotoarivony, Nicolas Mary, Farid Bahleman, Jörg Beckmann, Sandra Blome, Virginia Friedrichs, Horst Oebel, Hélène Jourdan-Pineau, Anjaranirina Marcel Mamiarisoa, Vincent Michaud, Johan Michaux, Christopher L. Netherton, Modestine Raliniaina, Tantely Randriamparany, Anissa Zouaoui, Ferran Jori Massanas

**Affiliations:** 1 UMR ASTRE, CIRAD, F-34398 Montpellier, France. ASTRE, Univ Montpellier, CIRAD, INRAE, Montpellier, France; 2 Department of Zootechnical Veterinary and Fish Farming Research (DRZVP), National Centre for Applied Research in Rural Development (FOFIFA), Antananarivo, Madagascar; 3 GenPhySE, Université de Toulouse, INRA, ENVT, Castanet Tolosan, France; 4 Program Transboundary Biosphere Reserve WAP Region, Deutsche Gesellschaft für Internationale Zusammenarbeit, Cotonou, Benin; 5 Nuremberg Zoo, Nuremberg, Germany; 6 Tapir and Suiform Taxon Advisory Group of the European Association of Zoos and Aquaria, Nuremberg, Germany; 7 Institute of Diagnostic Virology, Friedrich-Loeffler-Institut, Greifswald, Insel Riems, Germany; 8 Ecole Doctorale des Sciences de la Vie et de l’Environnement, University of Antananarivo, Antananarivo; 9 Conservation Genetics Laboratory, University of Liège, Liège, Belgium; 10 The Pirbright Institute, Pirbright, Woking, Surrey, United Kingdom; 11 National Veterinary Diagnostic Laboratory, Ministry of Livestock, Antananarivo, Madagascar; 12 Department of Zoology and Entomology, University of Pretoria, Pretoria, South Africa; University of the Faroe Islands: Frodskaparsetur Foroya, FAROE ISLANDS

## Abstract

In many rural areas of sub-Saharan Africa and Madagascar, domestic pigs (DPs, *Sus scrofa domesticus*) can coexist in sympatry, with species of the genus *Potamochoerus* spp. (*P. larvatus* or *P. porcus*). Reports of hybridization between domestic pigs and these two wild suid species are common in these areas. However, the existence and level of genetic introgression of those hybrids has never been sufficiently investigated to date to provide tangible conclusions on the occurrence of such interspecific hybridizations. The aim of our research was to investigate the genomic introgression of suspected hybrids following reported cross breeding events between DPs and *P. porcus* and *P. larvatus* in Benin and Madagascar, respectively. A total of 75 samples of individuals representing *Sus scrofa domesticus* (n = 32), *P. larvatus* (n = 7), *P. porcus* (n = 17) and putative hybrids (n = 19) were collected from the field and from European zoos. After filtering, 55 samples passed quality control and were retained for subsequent genotype analysis using 70K SNPs chip. The resulting genomic data was compared by principal component and admixture analysis. Our results revealed the absence of genetic introgression of *Potamochoerus* spp. in the selected putative hybrids. To further investigate these findings, we expanded our analyses to a global population of *Sus scrofa*, with the aim of identifying variations in their ancestral origins (Asian or European). These analyses revealed that the suspected hybrids from Madagascar and local DPs are derived from crosses between European and Asian DP populations, with proportions of 34% and 66%, respectively. In contrast, Beninese hybrids derived exclusively from European suids. This study provides, to our knowledge, the most comprehensive assessment to date of potential hybridization between DP and *Potamochoerus* spp. in sub-Saharan Africa. The great variability of local DP populations (European and Asian) may explain the presence of unique phenotypes when these distinct lineages are crossed, which may be falsely identified as hybrids between DP and *Potamochoerus* spp., if based on phenotypic data alone.

## Introduction

The African continent hosts several endemic genera of wild *Suidae*, notably *Potamochoerus*, *Phacochoerus*, and *Hylochoerus*. The genus *Potamochoerus* comprises two species: *Potamochoerus porcus,* red river hog and *Potamochoerus larvatus,* bushpig (BP) [1]. These two species differ significantly in terms of physical appearance, habitat preference and geographical distribution [2]. The BP is mainly found in East Africa, from Ethiopia to South Africa and Madagascar [2,3], while the red river hog inhabits the equatorial forest regions of West and Central Africa [4]. Genetic studies using mitochondrial and nuclear markers indicate that the BP was introduced to Madagascar, sharing similarities with populations from central southern Africa, particularly Zimbabwe [5]. Radiocarbon dating suggests its arrival in southwest Madagascar about 1,500 years ago, alongside domesticated animals such as zebu, sheep, and goats, with the first East African settlers [6].

Regarding the origins of domestic pig (DP) populations, the domestication of wild boar (*Sus scrofa*) approximately 9,000 years ago led to the emergence of DP, occurring independently in the Middle East and Asia [7]. These regions produced genetically distinct populations due to a 1.2-million-year divergence between their wild ancestors. The first domestication took place in Anatolia, Middle East, from where domesticated pigs migrated into Europe [8]. A second domestication event in Vietnam’s Mekong Valley led to the development of Asian pig breeds [9]. Today, DP are broadly classified into two major genetic lineages: European and Asian.

Concerning DP raised in Africa, Europeans introduced various DP populations to Africa between the 17th and 19th centuries. This led to the development of local breeds [10,11]. DP farming in Madagascar began in the 17th century with the introduction of Iberian breeds, locally known as ‘Kisoa Gasy’ [12,13]. Subsequently, other European breeds such as Craonnais, Bayeux, Berkshire, Tamworth, Large Black, Yorkshire Middle White and Large White were imported during colonization [14]. In West Africa, particularly in Benin, the local ‘West African dwarf pig’ is believed to have descended from European breeds and remains one of the most commonly reared breeds in the region [15,16].

Although African and Eurasian Suidae diverged from a common ancestor approximately 10 million years ago [17], suspected cases of hybridization between DPs and the genus *Potamochoerus* spp. have been repeatedly reported. These accounts emerge from various regions, including East and Southern Africa, as well as Madagascar for *P. larvatus* and in Central or West Africa for *P. porcus* (Table 1). Despite all these reports of hybridization, investigations from the genetic perspective to verify their occurrence have only been implemented occasionally.

**Table 1:** References mentioning hybrids between S. scrofa domesticus and Potamochoerus spp.

Species	Country/Region	Source
*P. porcus* & *P. larvatus*	Unspecific	[22]
*P. porcus*	Gabon	[23]
*P. porcus*	Unspecific	[2]
*P. porcus*	Benin	[24]
*P. porcus*	Benin	[18]
*P. porcus*	Unspecific	[4]
*P. porcus* & *P. larvatus*	Unspecific	[25]
*P. larvatus*	Kenya	[26]
*P. larvatus*	Uganda	[21]
*P. larvatus*	Madagascar	[27]
*P. larvatus*	Madagascar	[28]
*P. larvatus*	South Africa	[29]
*P. porcus* & *P. larvatus*	Unspecific	[30]
*P. larvatus*	Unspecific	[31]

In southern Benin a study was conducted to investigate the phenotypic diversity of DPs that are reared in this area [18]. This study identified individuals that were genetically distinct from local DP and selected breeds (cf Large White) by the analysis of 17 microsatellites. However, the design of this work did not include samples of *P. porcus*, making it difficult to confirm that the suspected hybrids were the result of hybridization between *P. porcus* and other DP. Additionally, the fixation index of population differentiation (Fst) between the hybrids and the two domestic populations (0.21 and 0.18) fell within the range typically observed between different breeds of DP [19,20]. Similarly, another study from Kenya, characterized the genetic diversity and genomic structure of local DPs [21]. This study strongly suggested the occurrence of hybrids between local DP and *P. larvatus* in rural areas of Kenya. However, these results were not clearly supported by Principal Component Analysis (PCA) and genetic clustering analyses. Therefore, to our knowledge, no study has confirmed or ruled out with sufficient certainty, the occurrence of hybrids between DP and *Potamochoerus* spp., despite numerous reports suggesting the observation of such hybridizations (Table 1).

The existence of such hybrids could have several implications in the investigation of solutions to mitigate the spread and impact of African swine fever virus (ASFV) in the world. Indeed, ASFV infection does not induce clinical signs of disease in BP [31–33]. Recent experiments have confirmed that *P. porcus* is equally resistant to ASFV infection (V. Friedrichs, personal communication). Hybridization could enable the inheritance of resistance traits from *Potamochoerus* spp., opening up a new avenue for research in the field of ASFV [31]. Therefore, this study aimed to investigate the genomic composition of suspected hybridization cases between *S. scrofa domesticus* and *Potamochoerus* spp. reported in three different populations of DP located in two rural locations in Madagascar and one in northern Benin. By clarifying the genetic ancestry of these animals, and considering that *Potamochoerus* spp. are resistant to the infection with ASF virus [33], we also expected to contribute to a broader understanding of the potential options of breeding hybrid pigs resistant to ASF virus as a potential approach for the management of this global disease [25,31].

## Materials and methods

### Site areas and population sampled

**Fig 1:**
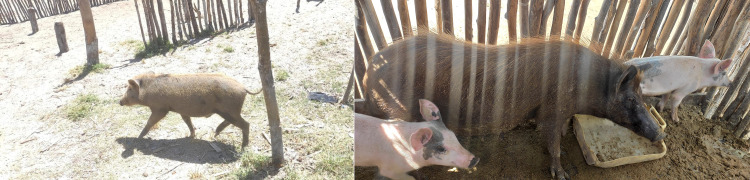
Examples of putative *S. scrofa domesticus* x *P. larvatus* hybrids suspected by local populations, Boeny (left) and Menabe (right) regions, Madagascar.

In Madagascar, the putative hybrids were located in two distinct west coastal regions, Menabe in the central west and Boeny in the northwest of the island (S1 Fig). Populations of free-ranging DP and *P. larvatus* are abundant in both regions, enabling their sympatric coexistence [28,34]. Previous deployment of participative approaches with local communities reported sympatry, including low levels of direct sexual interactions between both, DP and BP [27]. Farmers’ reports and observations of DPs displaying morphological traits resembling *P. larvatus* informed the selection of sampling sites (Fig 1). In addition, the sampling of potential hybrids was guided by local information, in particular reports of sexual interactions between domestic sows and BP males. Collaboration with local hunters facilitated the collection of tissues from BP samples.

**Fig 2:**
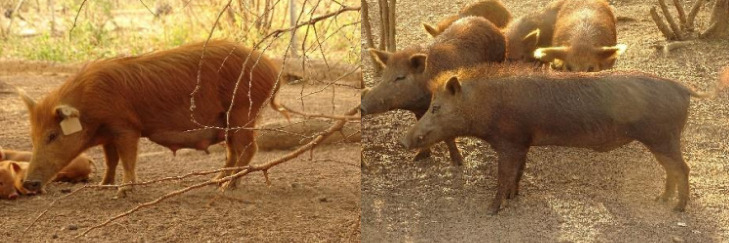
Examples of putative *S. scrofa domesticus* x *P. porcus* hybrids bred in Empire des Autruches farm-Atacora Region, Benin.

In Benin, we collected samples of a population of putative hybrids suspected to result from cross-breeding between a red river hog (*P. porcus*) and DPs kept in captivity at the Tatagtou farm in Dassari, Atacora department, northwest Benin (https://maisondestortues.org/wp-content/uploads/2023/12/Dassari.pdf). Those animals were openly marketed and sold as hybrids called “potamoporcs” [24] and several of them had atypical phenotypic characteristics compatible with hybrids of *P. porcus* (Fig 2) (S2 Fig). Our sample included tissues from 7 animals of different ages (ranging from eight months to seven years) resulting from these putative hybridizations.

The composition and provenance of all specimens included in this study are summarized in Table 2. The collected samples included ear cartilage and hair follicles preserved in absolute ethanol at room temperature, and extracts of organs such as liver, spleen and kidney stored at −80°C. A total of 75 samples were collected in the present study.

**Table 2:** Sample composition and provenance of studied suid populations, including wild species, DP, and putative hybrids.

Sample Type	Geographic Origin	No. of Samples	Source
Bushpig (*P. larvatus*)	Madagascar	7	Sampled in current study
Red river hog (*P. porcus*)	European zoos	17	Sampled in current study
Local Malagasy DP (DP_local)	Madagascar	18	Sampled in current study
Local Malagasy DP (DP_local)	Madagascar	14	Retrieved from ASTRE-Cirad repository (1998–2008)
Putative hybrid (HYB_larvatus)	Madagascar (DP × *P. larvatus*)	12	Sampled in current study
Putative hybrid (HYB_porcus)	Benin (DP × *P. porcus*)	7	Sampled in current study
**Total**		**75**	

### DNA extraction and genotyping

The NucleoSpin® Tissue Kit was used to extract DNA from tissues following the specified kit instruction involving proteinase K lysis and ethanol precipitation. Tubes containing 4 µl of DNA diluted to 50 ng/µl were prepared for genotyping. From hair samples originating from European zoos, total genomic DNA was isolated utilizing the DNeasy® Blood & Tissue Kit (Qiagen), following the manufacturer’s guidelines with minor modifications. In brief, five hairs that retained a follicle (with an additional 3 mm of hair) per sample were subjected to incubation in a lysis buffer for a duration of 6–7 hours at a temperature of 56° C. To ensure uniform lysis, the samples were agitated at a rate of 1,500 rpm. Following the complete lysis of hair keratin, the process of DNA extraction continued.

Genotyping was performed using the GeneSeek Genomic Profiler 70 K high density (GGP70KHD) porcine array (Illumina, USA) comprising 68,516 porcine SNPs (Single Nucleotide Polymorphism). Genotyping was undertaken by Aveyron Labo (Aveyron, France) according to manufacturer’s recommendations. The whole data set was produced on one beadchip. Genotypes were inferred from the raw fluorescence intensity data using the Genotyping Analysis Module included in GenomeStudio (version 2.0.5) software (Illumina, USA).

### Preliminary analysis of genotyping data, quality control

Quality control of SNP data was carried out using PLINK [35]. Individual samples were excluded if the number of missing genotypes were greater than 20%, while SNPs were removed if the missing genotype rate was greater than 20%. Since hybridization and other evolutionary factors like selection or genetic drift might cause a deviation from Hardy-Weinberg equilibrium (HWE), the HWE criterion was not considered for filtering variants. After quality control, 55 individuals and 52,642 SNPs were retained for genetic analysis (Table 3). The low sample retention rate (55/75) was attributed to two factors: prolonged exposure to ambient temperature prior to ethanol fixation (due to logistical difficulties in remote Madagascar field conditions), and the intrinsically low DNA yield from hair follicles.

**Table 3:** Population studied and sample size of suid populations after quality control.

Species	Label	Origin	Sample size
*Potamochoerus larvatus*	*P. larvatus*	Free ranging populations Madagascar	3
*Potamochoerus porcus*	*P. porcus*	Zoo populations from European Zoos	9
Putative hybrids *P. porcus* x *Sus scrofa domesticus*	HYB_porcus	Farm population, North Benin	7
Putative hybrids *P. larvatus* x *Sus scrofa domesticus*	HYB_larvatus	Farm population, Rural areas Madagascar	7
*Sus scrofa domesticus*	DP_local	Farm population, Rural areas Madagascar	29
**Overall**			**55**

### Population structure analysis

#### • Admixture.

To infer population structure, we used a maximum likelihood approach implemented in the ADMIXTURE software v1.3.0 [36] to detect the number of genetic populations (clusters or K). An unsupervised analysis was performed using the software’s default settings for K values ranging from 2 to 4, without any prior knowledge of the different ancestral populations. The optimal K value was estimated using a cross-validation procedure for each K value. The value retained corresponds to the lowest value.

#### • PCA analysis.

We performed a Principal Component Analysis (PCA) with snpgdsPCA function (SNPRelate package R) [37] to visualize the main groups of animals. The ggplot2 package [38] was used to visualize the results of these analyses.

To assess the ancestral origins (i.e., proportion of Asian and European suids) of our Malagasy DP, DP_local and putative hybrids, we retrieved genotyping data from previous studies on European and Asian suids [9,21,39]. Those genotypes were obtained using Porcine SNP60 (Illumina Inc.) or GGP70K chips and only the pure breed were retained. This dataset was merged with our samples (without wild African suids) by using PLINK [35]. The final dataset contained 21,387 common SNPs and 1,443 individuals (598 Asian_Suids, 802 European_Suids), 29 DPs from Madagascar (DP_local), 7 Malagasy putative hybrids (HYB_larvatus) and 7 putative hybrids from Benin (HYB_porcus) (S1 Table). PCA and a supervised Admixture analysis were performed on this new dataset. The supervised analysis was conducted by setting 2 reference populations:

i domestic and wild Asian suids (598 individuals),
ii domestic and wild European suids (802 individuals).

### Ethics approval

This study was conducted under the research permit N° 078/23-MEDD/SG/DGGE/DPRNE/SCBE.Re, granted by the Ministry of Environment and Sustainable Development. Ethical approval for this research study was granted by the Malagasy National Research Ethics Committee by the Ministry of Agriculture and Livestock with reference number 002–21/CENA. This study was approved by the Ethics Committee of the University of Abomey Calavi, Benin, under clearance n°0255–2022/UAC/SG/SA. Data were collected under the research permit n°417/MAEP/SG/DSV delivered by the Beninese Ministry of Agriculture, Livestock and Fisheries. All European Zoo hair follicles used in our study were collected during routine zoo inspections, which therefore did not require explicit authorization (in accordance with European Directive 2010/63/E7U).

## Results

### Local populations analysis

**Fig 3:**
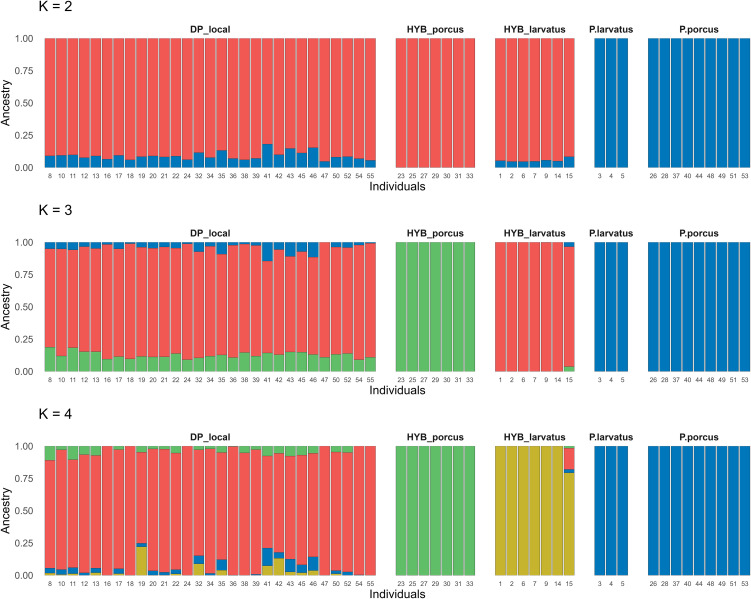
Admixture plot representing estimated membership coefficients for individual suids for ancestral populations (K) ranging between 2 and 4.

Admixture analyses ranging from K = 2 to K = 4 are shown in (Fig 3), which illustrated the genetic structure of the population under study. The lowest cross-validation values were obtained for K = 4 (S5 Fig) suggesting that K = 4 is the optimal number of clusters to characterize the genetic diversity within the sample set. Upon closer examination at K = 2, the analysis delineated two distinct groups: the first group consisted of Malagasy DPs (DP_local) and putative hybrids from Madagascar (HYB_larvatus) and from Benin (HYB_porcus), the second cluster correspond to the genus *Potamochoerus* spp. (Fig 3).

At K = 3, in addition to the separation of the wild and domestic groups, the HYB_porcus group also appears to be distinct. At K = 4, the HYB_larvatus group is clearly distinguished and separated from the other groups (Fig 3). However, at K = 4, one individual (ID n°15) from the putative hybrid group showed an ancestral composition with over 20% of local DP. This result suggests that this individual, while genetically affiliated with the HYB_larvatus group, carries a significant proportion of genetic ancestry shared with the local DP populations studied (DP_local).

**Fig 4:**
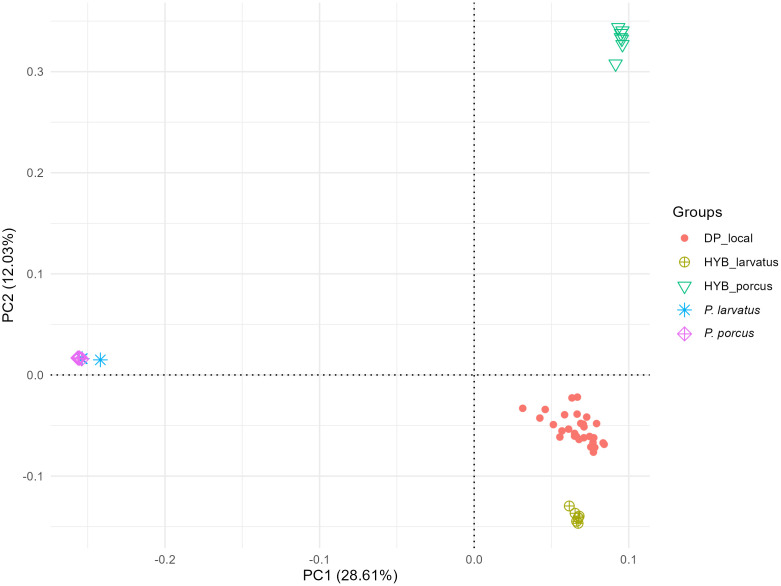
Population structure defined with PCA (PC1 and PC2) of 55 samples.

PCA revealed that Principal Component one (PC1) and Principal Component two (PC2) accounted for 28.61% and 12.03% of the total variance, respectively (Fig 4). The explained variance gain became very small from principal component 4 onwards (S3 Fig). PC1 allowed us to distinguish two groups of animals (*Potamochoerus* spp. and DP). The first group comprised by both African wild suids (*P. larvatus* and *P. porcus)*. The second group included DPs from Madagascar along with both groups of putative hybrids (from Madagascar and Benin). No individual or groups of individuals were found to be intermediate between domestic and wild clusters. The second and third PC allowed to differentiate the DP_local to putative hybrids groups (S4 Fig).

### Global populations analysis

**Fig 5:**
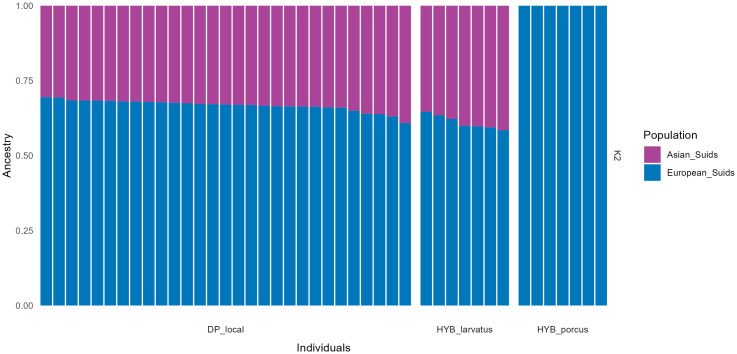
Supervised admixture analysis of DP (DP_local), putative hybrids from Madagascar (HYB_larvatus) and putative hybrids from Benin (HYB_porcus). Violet colour represents the ancestral proportion of Asian DP and wild boars (Asian_Suids) and blue the ancestral proportion of European DP and wild.

To investigate the different genetic backgrounds of local DP populations (including putative hybrids), similar analyses were carried out with the addition of Asian and European suids. Supervised admixture analysis using Asian and European suids as reference populations revealed a mixed genetic background within the DP population analyzed in our study (Fig 5). On average, individuals from Malagasy DP_local are characterized by 67% (± 2%) of their genome being of European origin and 33% (± 2%) of Asian origin. Similarly, the Malagasy putative hybrid population (HYB_larvatus) are characterized by an average of 61% European (± 2%) and 39% Asian (± 2%) ancestries. On the other hand, putative hybrids from Benin, HYB_porcus only showed a signal of European ancestry in their genome (Fig 5).

**Fig 6:**
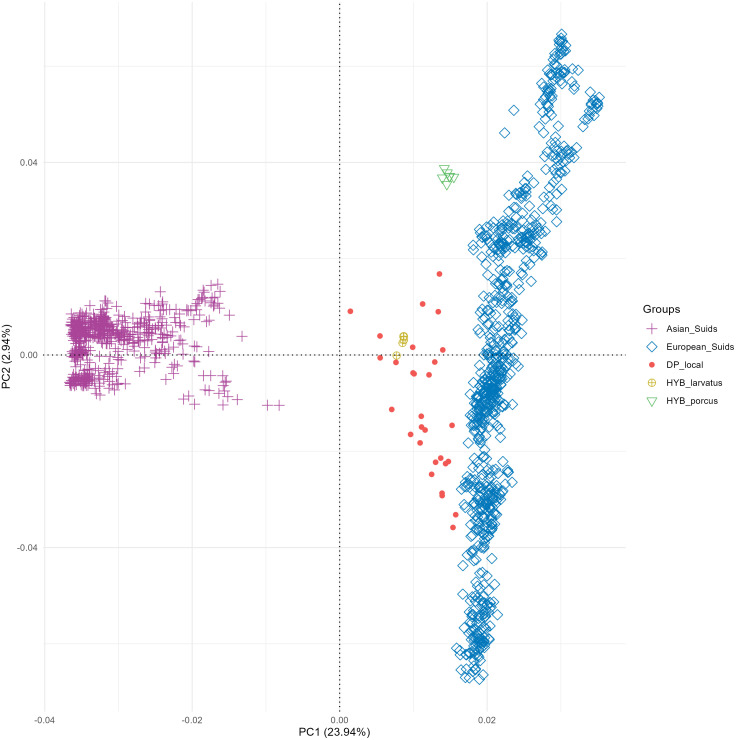
Population structure defined with PCA of the samples collected in this study (without African wild) in relation to global genotypes.

Principal component analysis revealed that the first dimension (PC1) accounted for 23.94% of the total variation, while the second dimension (PC2) explained 2.94%. The first principal component distinguished Asian suids from European suids. Additionally, PC2 mainly highlighted the variability among different European populations. Our samples, DP_local, HYB_larvatus and HYB_porcus occupied an intermediate position between the Asian and European populations (Fig 6).

## Discussion

Our study aimed to investigate suspected cases of hybridization between *Potamochoerus* spp. and *Sus scrofa domesticus* across three geographically distinct locations: two areas of Madagascar separated by 450 km and one farm located in Northern Benin, West Africa [25]. In Madagascar, suspicions of hybridization were triggered by the combination of particular phenotypes in habitats shared by domestic and wild suids under free-ranging conditions. It is worth mentioning that in all cases, mating between both species was only suspected and never witnessed directly by the informants. In Benin, the selective breeding of suspected cross-bred animals over several generations on a farm, known as “potamoporcs”, has promoted the idea of potential hybridization between *P. porcus* and DP. While some sampled individuals had atypical phenotypic characteristics compatible with the occurrence of potential hybrids (Figs 1 and 2), our results refute this hypothesis showing that such putative hybrids from the three distinct populations were not the result of *Potamochoerus* spp. and DP cross-breeds. Instead, these individuals likely resulted from the misidentification of atypical phenotypes arising from the genetic mixing of a wide range of pig lineages.

Building on prior studies, we used the GGP70 K HD Porcine array to genetically compare *S. scrofa domesticus*, *Potamochoerus* spp. and putative hybrids. By including both species of *Potamochoerus* (*P. larvatus* and *P. porcus*) our study provided optimal conditions to detect hybrids in contexts of sympatry and atypical phenotypes. We first assessed the genetic structure of our sample, including the two wild suids species and 29 DPs samples representing a subset of the local genetic diversity. The first principal component and admixture (K = 2) analysis revealed two clearly differentiated genetic clusters: one encompassing both *Potamochoerus* species, and the other comprising DPs, including both putative hybrid populations. This observation confirms the genetic proximity between the two species of *Potamochoerus* and a common ancestral origin [40]. Indeed, hybridization between *Potamochoerus* species have been reported in regions where their distributions overlap, notably in Uganda and Rwanda [2]. A recent study suggesting that the BP introduced to Madagascar could be the result of hybridization between *P. larvatus* and *P. porcus* supports this hypothesis [40]. However, neither PCA (PC1-PC3) nor Admixture analysis (K = 2 to K = 4) clearly distinguishes the two *Potamochoerus* species, despite their estimated divergence of around 300,000 years. This limitation likely stems from the SNP array’s design target: developed for *S. scrofa*, its markers may lack power to capture interspecific divergence at the genus level. Nevertheless, both PCA (PC1) and Admixture (K = 2) analyses robustly separate DPs from red river hogs and BP, indicating that the chip retains sufficient resolution to detect genetic divergence at the genus level and recent hybridization events.

Although we evaluated a limited number of populations, our results suggest that hybridization between *Potamochoerus* spp. and DPs is unlikely to occur under natural circumstances, refuting previous reports and observations drawn from phenotypes (Table 1). This result also questions the hypothesis of the potential viability of obtaining DPs naturally resistant to ASF virus as a result of cross-breeding between both species in natural conditions. Further studies should be encouraged by genetically testing additional populations of suspected hybrids or by attempting cross-breeding of both taxons in experimental or artificial conditions.

Beyond the lack of genetic proof, the long-term establishment of fertile hybrids would be fundamentally challenged by karyotypic differences. Indeed, the evolutionary separation between both genera has led to distinct genetic characteristics, including differences in chromosome numbers. The diploid number of chromosomes of DP (*Sus scrofa domesticus*) consists of 19 pairs of chromosomes (2n = 38) and is different from, red river hogs (*P. porcus*) and BP (*P. larvatus*) which encompass a karyotype of 17 pairs of chromosomes (2n = 34). These two genomes differ by the presence of two fusion chromosomes in *Potamochoerus* spp. that are homologous to pig chromosomes 13/16 and 15/17, as well as a rearrangement of chromosome 1 into an acrocentric form [41]. Although first-generation hybrids may appear chromosomally balanced, the fertility in such individuals appears more challenging to achieve. Indeed, such differences in chromosomal formula are known to result in genetically unbalanced gametes and non-viable embryos [42] that would explain the absence of hybrids.

Results of the PC2 and PC3 analyses, along with the admixture analysis for K = 4, indicated that DP_local individuals and both putative hybrid populations are genetically distinct. Given that the differences between the local pig population (DP_local) and the putative hybrids did not seem to be a result of hybridization with wild suid populations, we conducted a complementary analysis to understand the origin of these differences. To this end, we added genotyping data from global populations of *S. scrofa* and *S. scrofa domesticus*, including breeds from Asia and Europe. Principal component analysis revealed that the studied DP populations (DP_local, HYB_larvatus and HYB_porcus) exhibited an intermediate genetic composition between the two genetic backgrounds (Asian and European), with a genetic component more closely aligned to that of European DPs (Fig 6). To quantify the proportion of both genetic backgrounds, we performed a supervised Admixture analysis. The results revealed that Madagascar’s DP populations (DP_local and HYB_larvatus) have a similar genetic ancestry, with approximately 66% of European origin and 34% of Asian origin (Fig 5). These individuals may originate from breeds that were imported during colonization, including into Madagascar [12,43]. Indeed, between 18^th^-19^th^ centuries, crosses between Asian and European suids emerged in Europe, leading to the creation of synthetic breeds that significantly influenced the global evolution of DP breeds [44,45]. Alongside the introduction of European breeds, it is plausible that Asian breeds were also imported to Madagascar through trade arising from the Indian Ocean during the same period [14]. On the other hand, the same analyses revealed that DPs from the farm in Benin (HYB_porcus) encompass exclusively European genetic signatures. This suggests that these individuals may originate from European breeds not represented in our current dataset, or from indigenous breeds with predominantly European ancestry.

African DP populations exhibit ancestral diversity, stemming from multiple introductions of European and Asian lineages combined with local adaptation processes. This genetic variability contributes to a wide range of phenotypic expressions, particularly in traits such as skin and coat coloration [46]. For instance, in Nigeria, studies have identified various haplotypes of the MC1R gene, which influence pigmentation of indigenous pigs. For the Ashanti dwarf pig of Ghana, variations in pigmentation have been associated with genes such as SLC24A5, TYR, MITF, and TYRP1, whose alleles vary across ecological zones, suggesting adaptation to local environmental [47]. Such phenotypic diversity could potentially lead to misinterpretation as evidence of hybridization between *Potamochoerus* spp. and *S. scrofa domesticus*.

## Conclusion

Our study explored the occurrence of reported hybrids in the context of sympatry between *Potamochoerus* spp. and free ranging DP in three different pig populations involving one farm in Benin (West Africa) and two distinct rural areas in Madagascar where events of hybridization between *P. larvatus* and DP had been reported. The results of our genomic analyses challenge the potential existence of hybrids among our 14 suspected animals, as we found no evidence of *Potamochoerus* introgression in any of the samples collected from the three different populations analyzed in Madagascar and West Africa. Our genomic analysis suggests that hybridization between *Sus scrofa* and *Potamochoerus* spp. is unlikely to occur in natural circumstances. Based on important genetic diversity observed in local DP breeds, we hypothesize that potential reports of cross-breeding across the distribution areas of *Potamochoerus* in Sub-Saharan Africa and Madagascar are likely to be the result of misinterpretation. This false perception is likely driven by the combination of various factors: the morphological similarity between the two genera, their co-habitation in free-ranging conditions, and the occasional occurrence of atypical phenotypic traits in domestic swine populations, which can be produced by the crossing of diverse Asian and European lineages. Further studies of other cases of suspected hybridization in other locations are recommended to confirm our hypothesis.

## Supporting information

S1 Fig Distribution of pig farms where DPs and putative hybrids (HYB_larvatus) were sampled and where P. larvatus were captured. A (Boeny region, rural municipality of Bekipay), Madagascar. B (Menabe region, rural municipality of Befasy), Madagascar. The map was created by the authors using QGIS 3.10 a free opensource software http://qgis.org/. Base layer geospatial data (municipality boundaries, hydrography) are from the OCHA Common Operational Datasets (COD) for Madagascar (2018), (https://data.humdata.org/group/mdg) and OpenStreetMap (© OpenStreetMap contributors, licensed under ODbL) available on https://openstreetmap.org/ which are publicly available and unrestricted.
(PNG)

S2 Fig Location of the Tatagtou farm in the Atacora Department, Matéri Commune, Benin, where putative hybrids (HYB_porcus) were sampled. The map was created by the authors using QGIS 3.10 a free opensource software http://qgis.org/. Base layer geospatial data (municipality boundaries, hydrography) are from the OCHA Common Operational Datasets (COD) for Benin (2018), (https://data.humdata.org/group/ben) and OpenStreetMap (© OpenStreetMap contributors, licensed under ODbL) available on https://openstreetmap.org/ which are publicly available and unrestricted.
(PNG)

S3 Fig PCA: Proportion of the total variance explained by the first 10 principal components. (PNG)

S4 Fig Population structure defined with PCA (PC2 and PC3) of 55 samples. (PNG)

S5 Fig Cross validation plot indicating the model suitability as the number of putative populations (K) increases. (PNG)

S1 Table Sample populations merged with samples of DP_local and putative hybrids collected in this study (without African wild) in relation to global genotypes/ worldwide. (DOCX)
