# Supplementary figures and images for: Genomic analysis of putative hybrids between Potamochoerus spp. and domestic pigs from sympatric areas in West Africa and Madagascar
Source: PLoS One. 2026 Apr 21;21(4):e0346906. doi: 10.1371/journal.pone.0346906 (PMC13098969; doi:10.1371/journal.pone.0346906)

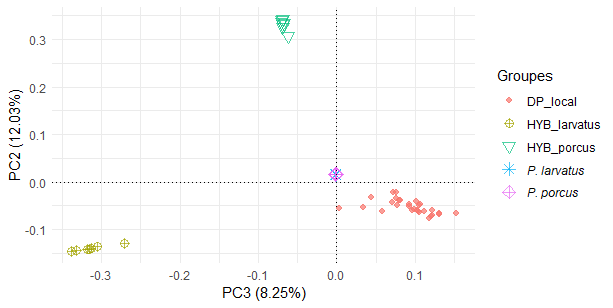

Supplement: S4 Fig — (PNG) [file pone.0346906.s004.png]

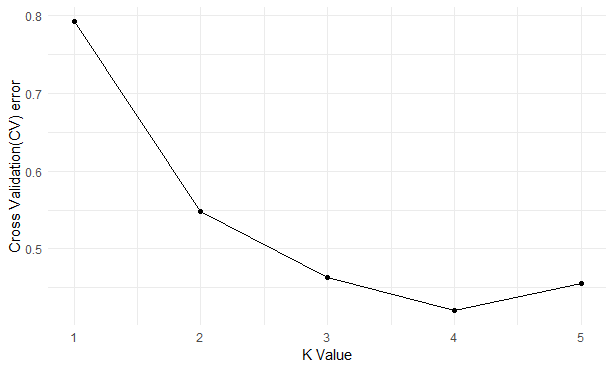

Supplement: S5 Fig — (PNG) [file pone.0346906.s005.png]
