# Supplementary material for: Genomic analysis of putative hybrids between Potamochoerus spp. and domestic pigs from sympatric areas in West Africa and Madagascar
Source: PLoS One. 2026 Apr 21;21(4):e0346906. doi: 10.1371/journal.pone.0346906 (PMC13098969; doi:10.1371/journal.pone.0346906)
Supplement: S1 Table — (DOCX) [file pone.0346906.s006.docx]

Supplementary table 1: Sample populations merged with samples of DP_local and putative hybrids collected in this study (without African wild) in relation to global genotypes / worldwide.

| **Group** | **Subgroup** | **Origin** | **Article** | **Number** |
| --- | --- | --- | --- | --- |
| Asian_suids | Asian wild boar | China | [1] | 29 |
|  | Asian domestic pig | China | [1] | 569 |
| European_suids | European domestic pig | China | [1] | 40 |
|  |  | Denmark | [1] | 36 |
|  |  | Germany | [1] | 40 |
|  |  | Hungary | [1] | 20 |
|  |  | Italy | [1] | 67 |
|  |  | Netherlands | [1] | 60 |
|  |  | Poland | [1] | 15 |
|  |  | Portugal | [1] | 15 |
|  |  | Spain | [1] | 67 |
|  |  | UK | [1] | 181 |
|  |  | USA | [2] | 204 |
|  | European wild boar | France | [1,3] | 37 |
|  |  | Italy | [1] | 16 |
|  |  | Poland | [1] | 4 |
| DP_local | Local Malagasy domestic pigs | Madagascar | Present article | 29 |
| HYB_larvatus | Putative hybrids *P. larvatus* | Madagascar | Present article | 7 |
| HYB_porcus | Putative hybrids *P. porcus* | Benin | Present article | 7 |
| **Overall** |  |  |  | **1443** |

**References**

1. Yang B, Cui L, Perez-Enciso M, Traspov A, Crooijmans RPMA, Zinovieva N, et al. Genome-wide SNP data unveils the globalization of domesticated pigs. Genet Sel Evol. 2017;49: 1–15. doi:10.1186/s12711-017-0345-y

2. Mujibi FD, Okoth E, Cheruiyot EK, Onzere C, Bishop RP, Fèvre EM, et al. Genetic diversity, breed composition and admixture of Kenyan domestic pigs. PLoS One. 2018;13: e0190080. doi:10.1371/journal.pone.0190080

3. Mary N, Iannuccelli N, Petit G, Bonnet N, Pinton A, Barasc H, et al. Genome-wide analysis of hybridization in wild boar populations reveals adaptive introgression from domestic pig. Evol Appl. 2022;15: 1115–1128. doi:10.1111/eva.13432
